# Current Hospital Topics

**Published:** 1906-06-02

**Authors:** 


### HOSPITAL ADMINISTRATION.

### CONSTRUCTION AND ECONOMICS.

### CURRENT HOSPITAL TOPICS.

#### Wanted: Annual Subscriptions.

It is to be wished that some steps could be taken whereby every great hospital in London would make a special effort to increase the amount of its annual subscriptions. St. George's Hospital used to hold the first place in regard to annual subscriptions, but of late years the amount raised has steadily fallen off until 1905 only yielded a sum of a little over £6,000 from this source. Of course the character of certain residential districts of London is constantly changing, but St. George's Hospital is situated upon a site in close touch with some of the largest and most fashionable squares in the West End, and there is no reason that we know of why every householder resident in the district should not be approached and, by adequate representation, induced to give an annual subscription every year to the hospital, which renders no small service to West End residents by admitting servants as in-patients. We venture to think that a little continuous and concentrated energy applied to the work of increasing the annual subscription list of every Metropolitan hospital must prove fruitful.

#### Artisans' Contributions to Hospitals.

An effort has been put forward lately in support of the General Hospital, Birmingham, with the object of increasing the proportions of the contributions made to its funds by those classes of the community which derive the most direct benefit from its work. It appears that the growth in the number of patients at the General Hospital shows a steady tendency to increase. Between 1867 and 1887 the patients treated in the Birmingham hospitals increased from 67,000 to 166,000 per annum, and since the latter year the number of patients has steadily grown. During last year no fewer than 66,935 patients were treated at the General Hospital alone. The actual sum given in the same year to this hospital by those classes of the community from which the patients are drawn, including the Hospital Sunday contribution, amounted to upwards of £6,000. The Hospital Saturday Fund in Birmingham raises upwards of £10,000 a year, a sum which is twice as much as that collected in Leeds, and three times as much as the artisans contribute to the Manchester hospitals. In London there is no doubt room for further effort to secure a more adequate contribution to the hospitals from the artisan classes. An indication of the sentiment of the working men towards our hospitals is afforded by the action of the workmen employed by the Pearson and Knowles Coal and Iron Company, who have agreed to raise £500 towards the £15,000 required to complete the extension of the Warrington Infirmary.

#### Hospital Book-keeping and Generous Donors.

We are glad to note that an attempt is being made to increase the interest of the annual reports of hospitals by giving a ground plan of the buildings and introducing illustrations showing features of the work, with the character of the furniture and fittings in the wards, theatres, and elsewhere. The Liverpool Royal Infirmary has made an advance in this direction in its 157th report just issued. We note, however, that the accounts are not kept upon the uniform system, a fact which is much to be regretted, having regard to the importance of this hospital, and the position it ought to hold among great provincial medical institutions. Liverpool has often shown considerable enterprise, and we venture to call the attention of Mr. Ralph Brocklebank, the treasurer, to this point in the hope that, in conjunction with Mr. Moore, the general superintendent and secretary, steps may be taken this year to place the accounts upon a modern basis by adopting the uniform system. This hospital had an adverse balance of upwards of £3,000 at the end of 1905, a fact which should make those immediately responsible active in support of a change in the form of accounts, because the larger contributors to hospitals nowadays do apportion their gifts, as we have reason to know, after some investigation of the form in which the accounts are rendered. Sound finance and good book-keeping have produced better financial results in regard to large hospitals in these days than almost anything else which can be mentioned. Now that the Royal Infirmary has been incorporated by royal charter, in view of the interest taken in the hospitals by H.M. King Edward VII., the treasurer will surely move in the direction indicated without a moment's avoidable delay?

#### The King's Fund and Hampstead General Hospital.

The King's Fund has promised a contribution of £5,000 to complete the £25,000 required to enable the whole of the buildings connected with the Hampstead General Hospital to be completed without delay. The proceedings at the annual meeting showed that at the present time the committee are £3,000 a year short of the income required to support the 64 beds and cots which the buildings at present contain. Should the further buildings be immediately proceeded with the present income will be at least £5,000 a year less than the amount required to maintain the completed hospital in efficiency. In such circumstances it would seem desirable that the committee should reconsider their position before proceeding with further buildings, for otherwise, as there are practically no invested funds, the committee may find themselves in a state of bankruptcy. In making their grant of £5,000 to the building fund the committee of the King's Fund Council expressed the hope that the medical staff would be strengthened by the appointment of honorary medical officers. At the present time the medical staff consists of local practitioners practising in the district, and as the hospital when completed will contain 100 beds it is essential in our view that the recommendation of the King's Fund should be promptly adopted. We regret to see that the local branch of the British Medical Association have passed a resolution in opposition to the recommendation of the King's Fund. We presume this step has been taken without adequate consideration, or a full appreciation of all the circumstances. We hope, therefore, that this recommendation will be withdrawn, otherwise the Hampstead General Hospital may find itself languishing for want of public support, unless the medical profession at Hampstead are prepared to provide the £5,000 a year additional income without which the enlarged hospital cannot be maintained.

